# The prevention access and risk taking in young people (PARTY) project protocol: A cluster randomised controlled trial of health risk screening and motivational interviewing for young people presenting to general practice

**DOI:** 10.1186/1471-2458-12-400

**Published:** 2012-06-06

**Authors:** Lena Sanci, Brenda Grabsch, Patty Chondros, Alan Shiell, Jane Pirkis, Susan Sawyer, Kelsey Hegarty, Elizabeth Patterson, Helen Cahill, Elizabeth Ozer, Janelle Seymour, George Patton

**Affiliations:** 1 General Practice and Primary Health Care Academic Centre, The University of Melbourne, Melbourne, Australia; 2 Centre of Excellence in Intervention and Prevention Science, Melbourne, Australia; 3 School of Population Health, The University of Melbourne, Melbourne, Australia; 4 Centre for Adolescent Health, Royal Children’s Hospital; Department of Paediatrics, Murdoch Children’s Research Institute, The University of Melbourne, Melbourne, Australia; 5 School of Nursing, University of Melbourne, Melbourne, Australia; 6 Youth Research Centre, University of Melbourne, Melbourne, Australia; 7 Division of Adolescent & Young Adult Medicine and Office of Diversity and Outreach, University of California, San Francisco, USA; 8 Centre for Health Economics, Monash University, Melbourne, Australia

**Keywords:** Young people, Health risks, Screening, Motivational interviewing, Emotional distress, Primary care, Health outcomes

## Abstract

****Background**:**

There are growing worldwide concerns about the ability of primary health care systems to manage the major burden of illness in young people. Over two thirds of premature adult deaths result from risks that manifest in adolescence, including injury, neuropsychiatric problems and consequences of risky behaviours. One policy response is to better reorientate primary health services towards prevention and early intervention. Currently, however, there is insufficient evidence to support this recommendation for young people. This paper describes the design and implementation of a trial testing an intervention to promote psychosocial risk screening of all young people attending general practice and to respond to identified risks using motivational interviewing. Main outcomes: clinicians’ detection of risk-taking and emotional distress, young people’s intention to change and reduction of risk taking. Secondary outcomes: pathways to care, trust in the clinician and likelihood of returning for future visits. The design of the economic and process evaluation are not detailed in this protocol.

****Methods**:**

PARTY is a cluster randomised trial recruiting 42 general practices in Victoria, Australia. Baseline measures include: youth friendly practice characteristics; practice staff’s self-perceived competency in young people’s care and clinicians’ detection and response to risk taking behaviours and emotional distress in 14–24 year olds, attending the practice. Practices are then stratified by a social disadvantage index and billing methods and randomised. Intervention practices receive: nine hours of training and tools; feedback of their baseline data and two practice visits over six weeks. Comparison practices receive a three hour seminar in youth friendly practice only. Six weeks post-intervention, 30 consecutive young people are interviewed post-consultation from each practice and followed-up for self-reported risk taking behaviour and emotional distress three and 12 months post consultation.

****Discussion**:**

The PARTY trial is the first to examine the effectiveness and efficiency of a psychosocial risk screening and counselling intervention for young people attending primary care. It will provide important data on health risk profiles of young people attending general practice and on the effects of the intervention on engagement with primary care and health outcomes over 12 months.

****Trial registration**:**

ISRCTN16059206

## **Background**

Particular terms and abbreviations used in this paper are defined under the heading ‘Defining Terms’ below.

### **Young people’s health and primary care**

The health of adolescents and young adults has received growing attention in Australia and worldwide [1-3]. Particular emphasis has been placed on the role of primary care services for 10–24 year olds, given that they comprise over one quarter of the world’s population and carry 15 % of the disease burden; 70% of premature adult deaths can be linked to risks beginning in adolescence [4]. Injury and neuropsychiatric problems are the greatest contributor to disease burden in these years[2], particularly with young people dying at a greater rate than any other age group from road accidents [5]. Other risky behaviours also emerge at this time, with unsafe sex and alcohol and tobacco use making a substantial contribution to disease in later life [1,3,6,7]. Risk taking behaviours and psychosocial morbidity tend to co-occur in individuals within this age group, [8,9] and are commonly associated with abuse and trauma [10]. Early detection and intervention for risk taking behaviours and mental health problems in the adolescent and young adult years have the potential to improve health during these years as well as preventing risks (e.g.: mental disorder, smoking, alcohol and drug misuse) for non-communicable diseases and premature deaths in adults [4,11,12].

These factors have highlighted primary care as an important setting for detection and early intervention of risk taking behaviours and mental health issues in youth. This is further supported by the frequency of visits, as most adolescents and young adults attend primary care at least once a year [13]. However, while this cohort has a high prevalence of emotional problems (36–40% of young people attending general practitioners have at least one emotional disorder) [14-16] young people with emotional and behavioural problems typically present to primary care with non-specific physical complaints rather than emotional concerns [17]. Furthermore, young people may not disclose their risk taking behaviours to health care providers unless prompted, in part due to fears about lack of confidentiality and being judged [13,18]. Yet in settings where they feel confident, young people welcome the opportunity to discuss health risks such as contraception, substance use and sexually transmitted infection with health care providers and trust their advice [19].

As a result, clinical guidelines have recommended discussing a range of prevalent health risk behaviours, mental health issues and safety concerns such as bullying and abuse with all young people, particularly where one health risk has been detected [20-23]. This approach is commonly seen as one aspect of developing effective and youth friendly primary health care [13]. Yet universal screening, particularly in the primary prevention of mental disorder in young people, remains controversial as there is little evidence about the clinical effectiveness and cost-effectiveness of screening programs [24].

### **Evidence for health risk screening in young people**

There has been a call for more evidence on the impact of universal health risk screening of young people on health outcomes, costs and potential harms [13,25,26]. In Australia, there is also very limited evidence for the role of practice nurses specifically in this work [27,28].

Two notable studies have tested screening for health risks during well visits [29,30]. In the first study, Ozer et al. tested the effectiveness of screening guidelines combined with preventive counselling and a comprehensive practice-based training system implemented in paediatric outpatient clinics in the US [29]. Detection and discussion of risky behaviours were increased with some suggestion of health benefits. Although most findings did not reach statistical significance, there were significant increases in helmet use with trends toward lower rates of risky behaviours across several other areas (tobacco, sexual intercourse, and non-use of seatbelts) reported in 15 year olds compared with the non-screened. The main limitation of this study was the lack of a longitudinal comparison arm.

The second study was a randomised controlled trial of preventive health counselling for teenagers having well visits with UK general practice nurses [30]. There was no difference in reported risk taking at 3 or 12 months. While there was positive movement along the stages of change continuum [31] at 3 months for diet, exercise and smoking, these changes were not sustained at 12 months. Other benefits included increased awareness of confidential health care access options and better mental health scores for those who were depressed [30].

There is evidence to suggest that detecting health risk in children and young people appears more effective during opportunistic screening compared with planned well visits [32]. This enhanced effectiveness may be due to a greater prominence of emotional and behavioural problems in acute care visits and because planned visits do not happen for all young people [32] or are more likely in those with fewer health risks. In Australia, opportunistic health promotion activity is more common because well visits for young people are not funded by the national health care system, Medicare.

### **Evidence for motivational interviewing as a primary care approach to health risk behaviours detected by screening**

Promising strategies for intervening with emotional and behavioural problems in young people have employed both cognitive-behavioural and motivational enhancement approaches [29,30,33]. Cognitive-behavioural strategies include education, advice, information about risk taking in peers, which is usually lower than young people think, and skills to refuse participation in risky behaviour [30,34]. Motivational enhancement interventions have shown promise in decreasing alcohol intake in young people [33,35]. The principles of motivational interviewing (collaborative, empathic, client-centred, supporting self-efficacy, rolling with resistance, addressing ambivalence, respecting autonomy) [36] fit well with the developmental stage of young people where there is commonly ambivalence about behaviours and frequently resistance to authoritative approaches towards change.

### **Addressing clinician barriers to detection of health risk and early intervention in primary care**

Barriers for general practitioners (GPs) in delivering this type of preventive care for young people include time constraints, lack of reimbursement and lack of training, skills and confidence in responding, plus the limited availability of appropriate referrals to specialist services [37,38]. Our recent work [39] and that of others [40] highlights that practice nurses (PNs) experience similar barriers to screening and counselling youth for health risk as GPs.

An educational intervention for GPs was developed by several of the authors to address these barriers [41] .This intervention used evidence-based strategies for changing clinician behaviour [42], and demonstrated that it was possible to improve the skills of GPs in communicating, and screening for health risks, with young simulated patients [41,43]. This intervention also improved the GPs’ self-rating of competency in youth health which was maintained five years post-training [44]. The intervention was underpinned by two theoretical perspectives: social cognitive (learning) theory [45] with a focus on improving self-efficacy, plus the theory of reasoned action [46] with a focus on altering behaviour by shifting attitudes and a perception of social norms.

As the same generic skills for screening and counselling young people for health risk are required across disciplines [47] and similar barriers to this are faced by PNs and GPs, we determined there was a rationale for adapting our educational intervention to target both PNs and GPs working with young people.

### **Addressing the evidence gap in health risk screening and early intervention in primary care**

We designed a pragmatic cluster randomised trial [48] to assess acceptability, effectiveness and economic efficiency of a universal, opportunistic, psychosocial health risk screening and motivational interviewing intervention for young people presenting to primary care. The motivational interviewing intervention was designed to train clinicians to respond to young people’s health risk behaviours in a non-judgemental way while still encouraging young people to contemplate change or adopt changes that would reduce health risks. We also designed a nested feasibility study to examine the role of nurses in screening and counselling youth. Additionally we explored the role of nurses in providing a point of linkage between the practice and other service providers who might collaborate in a care plan for youth issues requiring multidisciplinary input. We believe that this is the first ever trial to address these issues.

In this paper, we describe the development and piloting of the intervention and the protocol for the cluster randomised trial as well as variations in the implementation of the trial since trial registration. The detailed protocol for the economic evaluation and practice nurse feasibility study will be discussed in later outcome papers.

### **Policy relevance**

A steering group of relevant policy makers, practitioners and consumers was formed to ensure the project’s policy and practice relevance and ultimately to assist with feedback of results into policy and practice. Membership included: policy makers, GPs and PNs from medical and nursing associations and colleges, practice support staff (PSS) from pilot practices, the national peak body representing the coordinating regions for general practice (divisions), government departments of health (mental health, public health and primary care divisions), education and welfare representatives, young people and parent representatives.

### **Study questions**

The primary aims of the trial are to compare the intervention and comparison arms on: 

1. Clinicians’ accuracy in identifying risk-taking behaviour;

2. Young people’s uptake of risky behaviour or intention to change or reduction of established behaviour at three and 12 months post-intervention; and

3. Acceptability of risk screening to young people, their parents and practice staff.

The secondary aims include comparing both study arms on: 

1. Young people’s pathways to care, trust in their clinician and likelihood of returning for future visits;

2. Parents’ attitudes toward the concept of a youth friendly practice policy including seeing the clinician alone and conditional confidentiality.

The main study hypotheses originally reported in our trial registration [49] were that: 

1) An opportunistic screening tool for health risk in youth will improve the clinician’s detection by 25% compared to interview alone;

2) Specific risk response training, including motivational interviewing, will result in at least 10% overall less uptake of risky behaviour or greater intention to change or reduction in established risk behaviour three months post-intervention compared to usual care;

3) Any reduction in health risks would be sustained to 12 months;

4) The benefits for youth and society as a whole will outweigh the costs of the intervention; and

5) Youth preventive care and linkage role will be acceptable to PNs and general practice staff and will be feasible to implement.

We made three amendments to the hypotheses to overcome practical barriers to conducting the trial. The amendment to our first hypothesis, about the screening tool alone improving clinician detection, was made prior to patient recruitment, during the intervention phase of the trial. All intervention practices were given the screening tool and mentored in how they might integrate it with their office systems. However each practice differed in the way they used it (or whether they used it at all). Some preferred to use the desk top mnemonic chart we also provided to prompt screening. We therefore required flexibility in the way we implemented practice change, a characteristic of pragmatic trials [48]. Our first hypothesis was hence redefined to test whether the intervention training *and* screening tools will improve clinician detection.

The second amendment, made soon after recruitment of patients had begun, was to change the absolute percentage effect size shown in hypothesis one and two, to 31% and 15%, respectively. The minimum detectable effect sizes were modified in response to the slow recruitment rate of young people because the original sample size required to detect smaller effect sizes was unattainable within the time frame and resources available to conduct the study (see section on sample size estimates).

Hypothesis four, about the intent to conduct an economic analysis from a societal perspective, was amended during the study pilot to instead conduct the economic analysis from a health care perspective only. The societal evaluation approach requires the estimation of a monetary valuation of benefits. In addition to the large sample sizes required for such an analysis, there would have been a considerable responder burden as young people would already have been surveyed with a long interview for the main health outcomes.

## **Methods**

### **Intervention design**

**Figure 1:**
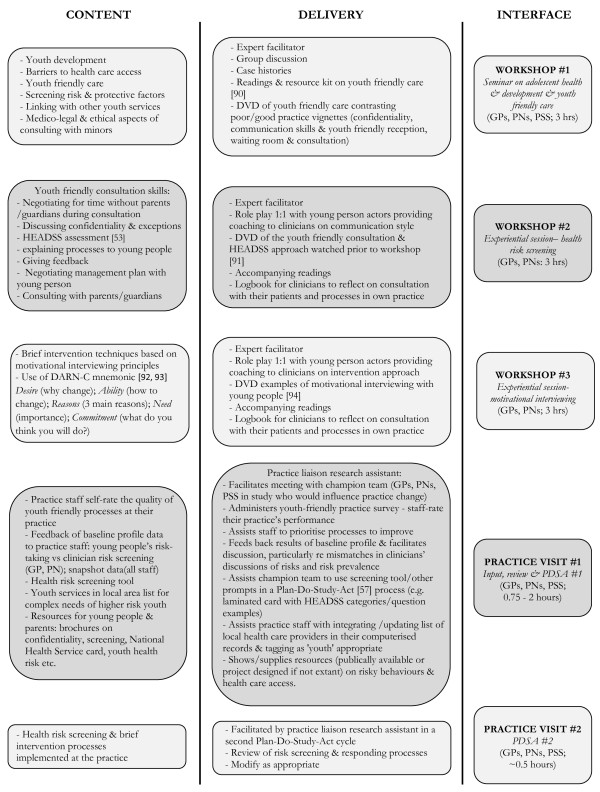
**Elements of PARTY 1 intervention and resources provided to practices.** Legend: GP = general practitioner; PN = practice nurse; PSS = practice support staff; PDSA = Plan-Do-Study-Act [78-82].

The intervention elements we developed are detailed in Figure 1.

Piloting of the intervention, measures and data collection for the trial were undertaken during the first year of the study (2005–6) in three pilot practices: an urban community health centre, an outer urban corporatised clinic and a rural private clinic [39]. The educational intervention for GPs designed in our previous work [41] was used in this study, adapted to fit a shorter time frame of nine hours.

Additional components were developed for this study:

a) Motivational interviewing (MI) training

A three hour workshop of MI training was developed using evidence-based principles about effective education and practice change [50] .The five general principles of MI were followed in designing the content of this component: expressing empathy, developing discrepancy, avoiding argumentation, rolling with resistance and supporting self-efficacy [36,51] .The approach included brief counselling options where there is minimal risk and longer options over 2 to 4 visits for moderate risk, in line with best evidence [34]. This training was delivered as an interactive workshop where clinicians practised ‘change’ discussions in role plays with young actors [41,52]. Accompanying resources included selected readings and a DVD made by the study team (HC and LS) demonstrating clinicians using an MI approach with young people in 4 vignettes (contraceptive use, tobacco use, marijuana use, and unsafe driving).

b) Screening tool and office systems

The screening tool was developed in consultation with GPs, specialists and young people based on the popular HEADSS mnemonic (Home: Education, eating, exercise; Activities and peers; Drugs, cigarettes and alcohol; Suicide, depression and other psychiatric symptoms and Safety) [53,54] and other existing screening tools [21,55]. The tool does not have predictive capability but preliminary doctoral work [56] indicates that a similar tool stimulates the desired discussion of risk-taking between the young person and their consulting clinician. The pilot was used to develop a process for assisting practices to resolve office system issues that would facilitate young people completing the screening tool including: site of completion (e.g., in a private room without parents present or in the waiting room); mode of completion (e.g., paper-pencil or on a personal digital assistant); storage of results and GP prompts for annual use.

c) Training (see Figure 1)

Training for GPs and PNs in the intervention arm involved three interactive workshops (three hours each) usually one or two weeks apart (facilitated by LS and HC) and two practice visits by research staff. PSS either attended the first workshop with clinicians or received this session separately. Training was intended to equip both GPs and PNs to: undertake opportunistic health risk screening of all young people presenting at the clinic (universal screening); and to provide health promotion advice and motivational interviewing based counselling to young people with identified health risk behaviours. To maximise participation in training, workshops were delivered at the practice or at a local venue where clinicians and PSS from several practices could attend.

d) Practice visits (see Figure 1)Within four to six weeks following the workshop training, researchers visited each ‘champion’ practice team (GPs, PNs, Practice Manager and receptionists) twice. The first visit began with practice staff completing a short survey rating the proficiency of their practice in various youth friendly characteristics. The survey prompted a discussion of how the practice might improve. The results of the baseline profile measures (see Table 1) of the practice snapshot of ‘youth friendliness’ and of the young people attending the practice were presented to staff. Young people’s self-reported health risks (and any intention to change) were graphed alongside the percentage of time clinicians screened for these health risks in the previous 12 months to highlight discrepancies between health risks, discussion of these risks, and missed opportunities for intervention. The results of the practice snapshot on youth friendliness highlighted practice systems working well and those needing improvement. This feedback was intended to provide practices with information about gaps in their current screening and youth friendly processes. The research team offered assistance in incorporating the screening tool into practice office systems following the model of continuous quality improvement ‘plan-do-study-act cycle’ [57]. The research team also provided practices with a limited range of other strategies to assist with youth friendliness (see Figure 1). The second visit, two weeks after the first, was to follow-up with the screening tool implementation processes.

**Table 1:** Measures developed for use in baseline, post-intervention and follow-up assessments

**Name of Measure**	**Nature of measure**	**Content of measure**	**Method of administration**	**Phase of administration**
*Practice measures*				
Practice snapshot	Proforma of qualitative questions and observer's field notes about practice	Organisational and staffing structure and youth friendly processes.	RA trained in assessing youth friendliness interviewed practice managers and directly observed practice (eg. Waiting room and materials). Detailed field notes recorded interactions with staff and incidents observed at the practice.	Baseline profile
*Staff measures*				
Staff survey	Likert scales and demographic survey	Self-perceived competency with youth friendly care and managing young people's health risk. Demographic data on age, gender and for clinicians, year of graduation, prior training in youth health or brief interventions and timing of these.	Self-completion written questionnaire	Baseline profile and post-intervention
Staff interview	Semi-structured interview	Acceptance of screening processes	Qualitative interview with RA, audio-taped	3-month follow-up phase
Nurse interview	Semi-structured interview	Feasibility of role in screening, counselling and linkage function	Qualitative interview with RA, audio-taped	3-month - 12 month follow-up phase
*Young people’s exit interview*				
Recall of Screening and Counselling [83]	Self-report categorical responses	Recall (occurring with healthcare provider) of screening/counselling for health risk	Computer Assisted Telephone Interview (CATI)	Baseline profile and post-intervention
GPAQ [84]	Self-report categorical responses			
Family Doctor Trust Scale [85]	Self-report categorical responses	Items related to trust in the clinician		
Likelihood of Future Visits Scale [86]	Self-report categorical responses	Items examining what conditions youth happy to see the clinician about		
Recall of other youth friendly processes	Self-report categorical responses	Confidentiality discussion, time alone with clinician		
K10 [87]	Self-report likert responses	Emotional distress		
Self-rating of mental and physical health [88]	Self-report categorical responses			
	SF12 [89]	Self-report likert responses	quality of life		
DEP-ADO [90,91]		Self-report categorical responses	Smoking, alcohol and other subtance use		
Abuse Screen [92]	Screening adolescents for abuse	Fear of a partner, fear of a family member for those 17 yrs and over			
Other risky behaviours/events	Self-report categorical responses and some likert scales	Unprotected sex, forced sex, road safety risk, eating and exercise Patterns, self harm, antisocial behaviour, bullying events.			
Considering risky behaviour change	Self-report binary yes/no response for self-identified risk				
Readiness for Change Ruler [93]	Self-report likert scales	Readiness for change			
Intention to Change [93]	Self-report catgeorical responses on a 5 point agree/disagree scale	Consideration of change			
Health service use	Self-report categorical responses	Use of a wide range of health services and costs			
*Young people's follow-up interviews*					
3 month follow-up and 12 month follow-up		Risky behaviour measures, K10 and SF12, abuse and violence measures as above; health service use since last consultation; whether had followed up on clinician's advice at initial consultation.	CATI interviewers	3 month follow-up and 12-month follow-up	
*Parent measures*					
Parent survey	Self-completion questionnaire: a mixture of categorical, likert and short answer questions	Parent opinion about various aspects of youth friendly care including health risk screening and confidentiality	Self-completion written questionnaire, sometimes completed over the phone by CATI if not returned in reply paid post.	Post-intervention	
*Consultation level measure*					
Clinician encounter form	Self-completion questionnaire: a mixture of categorical, likert and short answer questions	Reason for presentation, diagnosis, management.	Completed by clinician after consulting with a young person aged 14–24 years.	Baseline profile and post-intervention	

RA = research assistant; GPAQ = General Practice Assessment Questionnaire; K10 = Kessler Psychological Distress Scale.

SF12 = 12-Item Short-Form Health Survey; DEP-ADO = Detection of Alcohol and Drug Problems in Adolescents.

### **Comparison arm training**

To maintain engagement in the trial, GPs and PNs (but not PSS) in the comparison arm were offered standard training (three hour seminar) in young people’s health covering youth friendly care, screening for health risks, organising appropriate professional networks for management of complex issues and medico-legal aspects of consulting with minors. They were provided with a small selection of further readings. This basic training is already widely available for GPs in Australia. Whilst there was informal group discussion during the seminar, the delivery was in a Power-Point lecture format with no active learning through role-play or group work.

### **Study design**

We used a stratified cluster randomised trial to test the effectiveness of our intervention. The trial was organised according to the Extension of CONSORT statement to cluster trials [58]. General practices were the unit of randomisation rather than individual GPs because the intervention involved changes to the office systems and training of clinicians and PSS at the same clinic. Ethics approval for all phases of the study was obtained from The University of Melbourne Human Research Ethics Committee.

**Figure 2:**
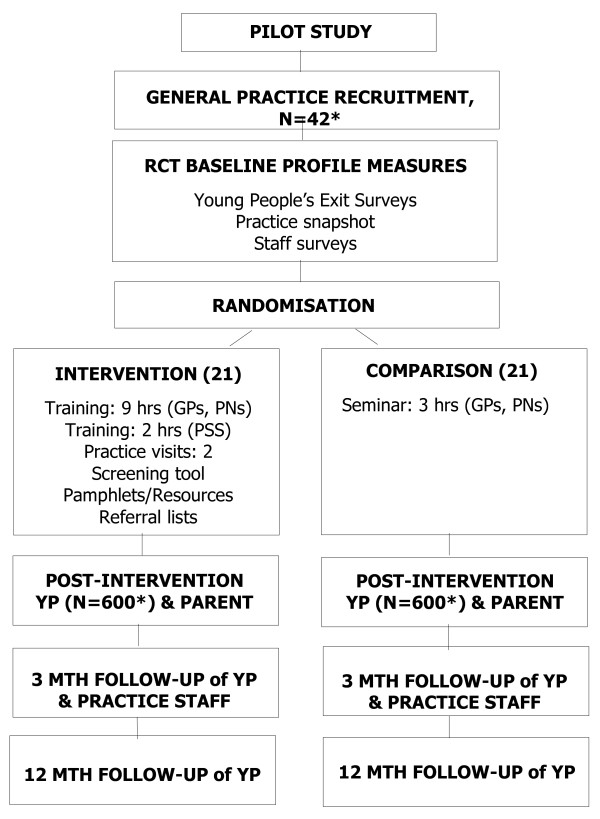
**Flow chart of PARTY Project design.** Legend: GP = general practitioner; PN = practice nurse; PSS = practice support staff; YP = Young people **.** *42 practices were to be recruited allowing for 2 to drop out and 1200 youth allowing for a 40% attrition over 12 months.

The overall trial flow and phases (year two-five, 2007–2011) are depicted in Figure 2. The description of the measures is provided in Table 1.

#### ***General practice recruitment***

We required 42 practices to be recruited from metropolitan and inner regional centres in the state of Victoria, Australia (see sample size calculation below). Initially our inclusion criteria for practices stipulated that a core group of staff from various disciplines within the practice (GPs, PNs, practice manager and receptionists) were willing to participate. Within the first few months it became apparent that this requirement was hindering recruitment as interested GPs took extensive time to consult with PSS who were often too busy to have interest in research participation. Hence we accepted expressions of interest from practices when at least one GP was willing to participate. This GP later involved the key PSS.

Initially practices expressing interest were required to see at least 25 young people aged 14 to 24 years per week to be included in the trial, so that recruitment periods could be kept at three weeks. This inclusion criterion proved a barrier to participation in the study of many practices that were interested but found it difficult to calculate the number of youth seen each week. To overcome the practical difficulty, we modified our requirement and accepted practices if they perceived young people to be a significant part of their patient base.

Practices were not required to have a PN because at the time of initiating this study only 40% of Australian practices had a PN [59]. Without a PN, it was intended that the linkage function would be provided by a GP.

A diverse range of approaches were used to recruit general practices including: 

•direct mail out and telephone calls to all GPs within general practice divisions in all metropolitan areas and 4 regional areas within 1–3 hours of Melbourne

•advertisements in newsletters for the Royal Australian College of General Practitioners (RACGP) and general practice divisions

•direct mail out to the Victorian General Practice Research Network [60]

•direct mail out and telephone calls to all practices in a Medicare Australia randomly generated list of 250 Victorian practices (200 urban, 50 rural) seeing at least 25 young people 14–24 years per week (Medicare Australia is the major federal government agency responsible for administering health care payments, services and policies and has data on these parameters for every practice).

Practices were offered the following incentives for participation: 

•practice payment of A$1000

•continuing education points for participating GPs (a RACGP requirement)

•feedback of their patient data in aggregated form

•opportunity to benefit from advances in the evidence base for youth primary care.

Practices expressing interest were visited by study team members to explain the study fully. Consenting practices signed Consent Forms and a Memorandum of Understanding outlining the roles and responsibilities of practice and research staff. Practices were briefed on the procedures for recruiting young people and collecting denominator data for young people seen during the recruitment periods as well as on the processes for the ‘practice snapshot’ and the staff and parent surveys (see measures outlined in Table 1).

#### ***Recruiting and interviewing young people***

Young people were recruited into the trial in the ‘baseline profile’ and the ‘post-intervention’ phase (see Figure 2). The ‘baseline profile’ was collected prior to the randomisation of the practices to provide a snapshot of the young people seen by the clinician and to assess for baseline differences between the study arms after randomisation. The post-intervention sample of young people was a different sample from the baseline sample and became the study cohort followed up at 3 and 12 months.

a) Inclusion/exclusion criteria

All youth aged 14–24 years attending participating clinicians were eligible for participation. Exclusion criteria were: being physically or mentally (very) unwell (vomiting, febrile, weak, cognitively impaired or psychotic); unable to read or speak English; being under 18 years of age AND unable or unwilling to obtain parental consent AND judged by the clinician as incompetent to make an informed decision for participation in minimal risk research (that is, an immature minor) [61].

b) Recruitment and interview

Recruitment of young people into the study occurred in two phases. First, clinicians were trained to ask all eligible young people, after their consultation had finished, whether they would be willing for their contact phone number to be passed on to researchers for a full explanation of the study. GPs or PNs recorded telephone numbers of interested youth and any reasons for refusal or ineligibility; the clinicians were asked to fax this list to the researchers daily. The computer assisted telephone interview (CATI) staff telephoned all listed youth (usually on the same day) to fully explain the study and obtain formal consent. CATI staff then conducted the exit interview with consenting young people or made an appointment for the interview to take place at a time convenient for the young person. The interview (approximately 50 minutes long) could also be completed over several phone calls if not finished in one sitting. Young people were first notified by Short Messaging Service (SMS) to alert them that a call was coming from the project team. At the end of the interview, CATI staff obtained consent to telephone young people for the three month and 12 month follow-up periods. Follow-up interviews were approximately 20 minutes long. Young people had up to 20 telephone call attempts for up to two weeks before they were recorded as a missed recruit.

Young people, who completed a telephone interview, were given the option of entering a draw for an iPod valued at A$200 (drawn after each 500 interviews).

The data manager checked the numbers of youth recruited into the study against the total number of attendances at the practice that day, after retrieving the best possible information on this denominator figure from the practice software systems or manual counts. If the numbers of young people clinicians approached fell short of attendances, practices were phoned to offer assistance to improve recruitment.

Obtaining the total number of attendances at each practice each day was difficult because the typical appointment and consultation software used by practices is not designed to extract this information; many computer systems crashed when queries were run. Furthermore, rather than recording individual young people attending the practice, most appointment software systems capture the number of encounters, which may include one individual twice or more (e.g., when someone presents for a review). In addition, many nurse visits were not recorded electronically by practices.

The difficulty determining the total number of presenting patients over the data collection period is important in determining the representativeness of the study population compared to the whole population attending the participating clinicians and, thus, exactly how many young people might have been exposed to the intervention. The latter estimate is important for the economic evaluation. Sensitivity analysis will be conducted to explore the impact this may have on the findings of the study.

#### ***Baseline profile of young people***

Prior to randomisation of the practices, clinicians were instructed to recruit all eligible young people aged 14–24 years whom they saw within the two weeks baseline profile period. Clinicians completed a clinician encounter form (see Table 1) for each youth consult.

#### ***Baseline snapshot of staff and practice***

After baseline sampling of young people, the practice staff (participating GPs, PNs and PSS) were administered the staff survey and the research assistant conducted the practice snapshot visits. The purpose of this phase was to describe the baseline youth friendly characteristics of the practice; the self-perceived competency of participating staff in dealing with young people; and, to understand the way innovation is adopted within the practice.

#### ***Post-intervention sampling of young people and parents***

Eight weeks after randomisation and six weeks after the practice staff completed the intervention, practices recruited the young patients who formed the cohort followed up at three months and 12 months post-consultation. Clinicians were again instructed to recruit all eligible young people aged 14–24 years with whom they consulted until the required sample size for each practice was achieved. Parents attending with young people under the age of 18 years were instructed to complete the written parent survey and return it to researchers in a reply paid envelope. Clinicians again completed a clinician encounter form on each youth consult.

Our monitoring of recruitment rates of young people revealed that clinicians were failing to recruit continuously, and systematically; the timeframe for recruitment extended over long periods that varied from 13 days to nearly 11 months (mean 86.3 days). Within the longer periods, recruitment was inconsistent, influenced by practice constraints (e.g., accreditation activities, staff absence including extended sick leave as was the case for the practice which took 11 months). To complete the study within a reasonable timeframe and to ensure that all eligible young people were approached and recruited into the study, we changed our recruitment strategy for the last 15 practices by placing a research assistant in the waiting room to systematically approach every young person with an invitation to be involved. Slightly more intervention practices (8/19 intervention versus 7/21 comparison practices) received this assistance because recruitment in the intervention practices was delayed after randomisation due to longer times taken for training clinicians compared to the comparison practices. The additional benefit of the in-practice research assistants was the timely, daily transfer of phone numbers of eligible young people to the CATI interviewers and accurate recording of reasons for refusal to participate or ineligibility.

#### ***Follow-up at three and 12 months (year two-five)***

Youth from the post-intervention sample were called by CATI staff for their three month and 12 month follow-up interviews. The three month interviews were completed by November 2010 and the 12 month interviews were completed by the end of July 2011.

#### ***Post-intervention interview with practice staff***

As part of our process evaluation, semi-structured interviews with GPs, PNs and PSS were undertaken for both study arms three months post-intervention to determine the acceptability of their respective screening methods and whether any practice changes were sustained. The clinician encounter forms on each patient consultation along with the young person’s exit interview also contain data that will capture the extent to which the intervention was implemented as intended.

#### ***Randomisation***

The randomisation of practices was stratified by postcode level advantage-disadvantage scores (SEIFA - Socio-Economic Indexes for Areas) for Victoria in 2001 [62] (dichotomised into low versus middle/high tertiles) and type of practice (private billing, bulk billing, and community health centres) forming 6 strata. Bulk billing practices do not charge patients for the consultation but accept only the fixed national health care rebate for the consultation specified by the Australian government. These strata were chosen because young people from lower socio-economic areas are more likely to consult bulk-billing practices and are more likely to have psycho-social health risks [5]. Block randomisation with fixed block sizes of two was used within strata. Randomisation was carried out by an independent statistician not connected with the trial. The independent statistician informed the project manager (BG) of the randomisation result and the practices were informed after all their baseline profile data collection was completed. The allocation sequence remained concealed from the research team until all practices entering the trial had their interventions assigned.

#### ***Blinding***

Due to the nature of the intervention it was not possible to blind the practices to their study arm status. The CATI staff and the in-practice research assistants who recruited patients into the trial were blind to the study arm status of the practices. Young people were not informed of the intervention status of their practice in any written or verbal communication from the researchers. It is unknown whether practice staff may have inadvertently informed patients about the study arm status of the practice.

#### ***Sample size estimation***

Sample size calculations assumed 80% power at a significance level of 5% for two- sided test and an intra-clinic correlation of 0.04 [63]. The original first hypothesis was based on the assumption that 40% of youth attending general practice have health risk behaviours or emotional distress, and that trained GPs, at best, will pick up 60% with interview alone [17], equivalent to 24% of all presenting youth. For a clinically meaningful outcome, we expected the clinicians to detect a further 25% of youth with risk taking behaviours using the screening tool with the interview (intervention arm), equivalent to an additional 10% of all presenting youth to the practice, compared to the comparison arm. To detect a 10% difference in the clinician’s detection of risk taking behaviours of all youth attending general practice between the two study arms, 1760 youth (44 per practice in 40 practices) were required after allowing for a variance inflation factor (VIF) of 2.72.

To allow for the slow recruitment rates, we revised our intended intervention effect in the clinician’s detection of risk taking behaviours of all youth attending general practice from 10% to 12.5% (equivalent to intervention clinicians picking up a further 31% of youth with risk taking behaviours instead of the 25% originally proposed). This small adjustment to the effect size reduced the required sample size to a total of 720 youth (18 per practice in 40 practices with a VIF of 1.68). To allow for a 40% loss to follow-up of youth over 12 months [64], we calculated that a total of 30 youth in each practice needed to be interviewed at recruitment. An additional two practices were also recruited to allow for loss of practices during follow-up.

The revised sample size also affects the difference in the prevalence of the risk behaviours that can be detected between the study arms in the second hypothesis. Table 2 gives the minimal effect size that can be detected in the prevalence of selected risk behaviours between the study arms at 3 and 12 months follow-up (power 80%, alpha 5% for a two-sided test) for the revised sample size for a range of intra-cluster correlations. The revised sample size is sufficient to detect at least a 15% difference between the two study arms for each of the risk behaviours with 80% power and alpha at 5% for a two-sided test.

**Table 2:** Minimal effect size that can be detected for the revised sample size given comparison arm risk behaviour prevalence, for a range of ICCs*

		**Effect size**		
**Risk behaviours**	**Control**[7]	**ICC = 0.01**	**ICC = 0.04**	**ICC = 0.07**
Alcohol use	41%	11%	13%	15%
Substance abuse	38%	11%	13%	15%
Tobacco use	24%	9%	10%	12%

ICC = Intracluster Correlation Coefficient; Control = comparison arm.

*Calculations assume an 80% power with alpha of 5% for a two-sided test.

#### ***Measures developed***

Outcome measures were developed for baseline, post-intervention and follow-up at three and 12 months. Data were collected from five sources: the practice, practice staff, young people and parents (if accompanying the young person) and the clinicians’ reports of the consultation in the clinical encounter form. The measures used to collect the data are detailed in Table 1. Phase of administration refers to phase of the trial flow detailed in Figure 2.

### **Data analysis plan**

Quantitative outcomes: Descriptive statistics will be used to compare clinician and young person’s characteristics between the two study arms at baseline and measures taken immediately after the consultation. Intra-cluster correlation will be reported for the main outcomes. Analysis will be by intention to treat and results will be reported following the extended consort statement for cluster randomised trials [58]. Marginal linear and logistic regression models using generalised estimating equations with robust standard errors will be used to adjust for the clustering effect of randomising the general practices and outcomes measured at the young person’s level. Marginal logistic regression will be used to estimate the intervention effect for binary outcomes and will be reported as odds ratios. Marginal linear regression will be used to estimate the intervention effect for continuous outcomes and will be reported as the difference in the mean outcome between the two study arms. Estimates of the intervention effect will be reported with their respective 95% confidence intervals and p-values. Multivariable regression models will be used to adjust for variables used to stratify randomisation (SES of the practice location, practice billing type) and for imbalanced baseline factors identified a priori to be strongly associated with the outcome, namely the baseline risk factor status of young people and prior training in adolescent health. Recruitment method of young people in the trial will also be adjusted for in the regression model (clinician versus research assistant).

Qualitative outcomes: Interviews with practice staff on acceptance of screening processes will be transcribed and analysed for themes. Secondary outcomes of pathways to care (health service use) [65] will be described and parental attitudes will be summarised for each arm.

### **Economic evaluation**

The economic evaluation will be undertaken from a health care perspective. Costs include the resource use required to develop and administer the intervention, changes in clinical practice associated with the initial GP or PN consultation and the young person’s subsequent health service utilisation during the follow-up period. Health outcomes consist of the changes in risky behaviours measured in the main trial and health related quality of life using the SF-12 [66]. The incremental costs will be compared to the incremental benefits in a cost consequence analysis. If the intervention proves to be effective at reducing specific risk behaviours then a cost-effectiveness analysis will also be reported. Finally, if the intervention is successful in improving quality of life (as reflected in the SF-12 data and the associated SF-6D quality of life tariffs), then a cost-utility analysis will also be reported [67].

## **Discussion**

The strengths of this trial of a multi-faceted intervention in a complex system for a hard to reach population (youth 14–24 years) include its cluster RCT design with follow-up at 3 months to assess the effect of the intervention on the health outcomes of young people and at 12 months post-intervention to assess the sustainability of these health outcomes.

Most patients consulting GPs and PNs have comorbid conditions [68] and young people are no exception [8,69]. We therefore chose a pragmatic trial for testing the potential of the recommended practice of screening young people (aged 14–24 years) for multiple health risks [20,70] and for providing an appropriate response to make a difference to health outcomes.

This study is the first reported randomised trial protocol of a universal, opportunistic health risk screening and counselling intervention for young people presenting to primary care. This trial will provide additional evidence of acceptability to young people, parents, GPs, PNs and PSS. Furthermore, this trial measures changes in clinician behaviour and also health outcomes for young people. Trials of training interventions that evaluate both clinician change and patient health outcomes are still uncommon [71]. The training delivered to clinicians to equip them to respond to young people’s health risk was brief to enhance uptake by primary care clinicians who are time poor so is realistic for the primary care setting. Few behavioural screening and intervention studies of youth in primary care have an economic evaluation [25]; our trial will be helping to address this gap.

There are also few studies describing the feasibility of the PN role in youth preventive and early intervention primary care, particularly in an Australian setting. Our results will directly inform policy about the PN’s role in screening and preventive counselling as well as linkage with other primary care, secondary care (medical and mental health), and welfare, education and justice organisations.

As expected when conducting a trial in the real world setting, some changes to the protocol after trial registration, were necessary to overcome practical constraints in conducting the trial. These constraints arose because of the sheer busyness of general practice which prevented staff from adhering to some of the original research protocol, as well as the lack of functionality in practice software to enable accurate, easy collection of data on the number of presenting patients in each practice per day [72].

There is a possibility that unsystematic clinician recruitment could have introduced a recruitment bias, although this problem would occur in both arms of the trial. There is also a possibility of systematic bias or differential recruitment [73] in the two study arms because young people were recruited after practices were randomised and were therefore approached by clinicians who knew their study arm status. It was not possible to recruit patients prior to randomisation of practices because the intervention being tested was opportunistic screening for, and response to, health risks for a young person presenting for any reason to their clinician on the day. In addition, the alternative of RAs recruiting patients instead of the clinicians carried prohibitive costs when we started this trial. We tried to circumvent this risk of differential recruitment by asking clinicians to record information on all young people seen and by having CATI staff (blinded to study arm status) actually obtain the consent and conduct the interviews. The change in recruitment strategy to in-practice RAs, three quarters into the trial, will be adjusted for in our final analyses as the type of young people recruited by each method may be different.

The modifications to the inclusion criteria, namely, accepting practices that saw less than 25 young people seen per week and practices where the majority of the clinicians in the practices need not be involved in the study, considerably increased the time needed to recruit the minimum number of young people from each practice required to test our hypotheses, particularly the practices that did not meet the initial inclusion criteria. This extended the anticipated study timeline by an extra year. The practical difficulties of recruiting young people, and the extension of the recruitment period, increased both the research burden on practice staff, the study length and research costs. Revision of the sample size was therefore necessary to counterbalance the practical difficulties, time and cost of conducting a large scale and pragmatic trial of a complex intervention [74].

Our difficulties with trying to implement changes in screening at the practice level within a relatively short time frame of four to six weeks highlights the need for trials of youth friendly interventions that tackle the practice system as a whole over much longer time periods. Evaluation of changes in practice organisation is required over longer time periods and at multiple time points to capture evolving change. Longitudinal studies of organisational change are infrequent, yet necessary to understand if interventions are to be appropriately designed to facilitate adoption [75]. Our experience also reinforces that a standardised intervention in complex systems, such as general practice, cannot be uniformly implemented as each practice has unique drivers, enablers and constraints [76,77].

Our analysis of the results of this trial is currently underway and will provide an important contribution to the literature on preventive interventions for youth attending primary care.

## **Defining terms**

‘clinician’

general practitioner (GP) or practice nurse (PN)

‘practice support staff’ (PSS)

refers to practice managers and receptionists

‘young people’ and ‘youth’

refer to the age group under investigation in this study (14–24 years) which also includes ‘adolescents’ (aged 14–19 years).

‘screening’

refers to a discussion of health risks between a young person and clinician or use of a screening tool to detect health risks.

‘opportunistic’

refers to screening that the clinician conducts with young people during the course of a consultation which the young person has initiated for any reason

‘well visit’

when the young person attends primarily for a health promotion or preventive health service.

‘universal’

describes screening and counselling approaches for all young people presenting to the clinician.

## **Competing interests**

The authors declare that they have no competing interests.

## **Authors’ contributions**

LS conceptualised the research with input from GP and SS. LS, SS, GP, AS, JP, KH, EP and PC developed the study design with input from EO. LS conceptualised the pilot preparatory work. LS and BG oversaw the running of the trial. AS and JS advised on, and JS implemented, all economic aspects of the trial. LS and HC designed the intervention with input from EO and LS and HC delivered the intervention training. LS and BG drafted the protocol. All authors read and edited successive drafts and approved the final manuscript.

## Pre-publication history

The pre-publication history for this paper can be accessed here:

http://www.biomedcentral.com/1471-2458/12/400/prepub
